# Performance of an automated ultrasound device in identifying and tracing the heart in porcine cardiac arrest

**DOI:** 10.1186/s13089-021-00251-5

**Published:** 2022-01-03

**Authors:** Paul Olszynski, Rory A. Marshall, T. Dylan Olver, Trevor Oleniuk, Cameron Auser, Tracy Wilson, Paul Atkinson, Rob Woods

**Affiliations:** 1 Department of Emergency Medicine, University of Saskatchewan, 103 Hospital Drive, Saskatoon, SK S7N 0W8 Canada; 2 Western College of Veterinary Medicine, University of Saskatchewan, Saskatoon, Canada; 3 College of Medicine, University of Saskatchewan, Saskatoon, Canada; 4 College of Arts and Sciences, University of Saskatchewan, Saskatoon, Canada; 5 Department of Emergency Medicine, Dalhousie, Saint John, Canada

**Keywords:** Cardiopulmonary resuscitation, Echocardiography, Artificial intelligence, Automation, Image segmentation, Cardiac arrest

## Abstract

**Background:**

While intra-arrest echocardiography can be used to guide and monitor chest compression quality, it is not currently feasible on the scene of out-of-hospital cardiac arrests. Rapid and automated sonographic localization of the heart may provide first-responders guidance to an optimal area of compression without requiring them to interpret ultrasound images. In this proof-of-concept porcine study, we sought to describe the performance of an automated ultrasound device in correctly identifying and tracing the borders of the heart in three distinct states: pre-arrest, arrest, and late arrest.

**Methods:**

An automated ultrasound device (bladder scanner) was placed on the chests of 7 swine, along the left sternal border (4th–8th intercostal spaces). Scanner-generated images were recorded for each space during pre-arrest, arrest, and finally late arrest. 828 images of the LV and LV outflow tract were randomized and 150 (50/state) selected for analysis. Scanner tracings of the heart were then digitally obscured to facilitate tracing by expert reviewers who were blinded to the physiologic state. Reviewer tracings were compared to bladder scanner tracings; with concordance between these images determined via Sørensen–Dice index (SDI).

**Results:**

When compared to human reviewers, the bladder scanner was able to identify and trace the borders during cardiac arrest. The bladder scanner performed best at the time of arrest (SDI 0.900 ± 0.059). As resuscitation efforts continued and time from initial arrest increased, the scanner’s performance decreased dramatically (SDI 0.597 ± 0.241 in late arrest).

**Conclusion:**

An automated ultrasound device (bladder scanner) reliably traced porcine hearts during cardiac arrest. It is possible a device could be developed to indicate where compressions should be performed without requiring the operator to interpret ultrasound images. Further investigation into rapid, automated, sonographic localization of the heart to identify the area of compression in out-of-hospital cardiac arrest is warranted.

## Introduction

Survival for out-of-hospital cardiac arrest (OHCA) remains low at approximately 10–15% [1–3]. Chest compressions during cardiopulmonary resuscitation (CPR) play a central role in survival. According to the CPR guidelines of the American Heart Association, compressions should be performed midline over an area of compression defined by the lower half of the sternum [3]. During compressions, forward blood flow results from a combination of effects on the thoracic cavity and heart, generating flow through what are postulated as the thoracic and cardiac pumps, respectively [4–6]. However, emerging research suggests that compressions over the current landmark often result in outflow obstruction, significantly limiting or even compromising pump effect [7–11]

Studies and reports involving the use of transesophageal echocardiography (TEE) during cardiac arrest have demonstrated that moving the area of compression lateral to the sternum can alleviate aortic outflow obstruction and improve left ventricle (LV) compression [12–17]. However, TEE is not currently feasible at the scene of OHCA. The use of transthoracic echocardiography (TTE) to identify the optimal area for LV compression and monitor chest compression quality has also been described with associated improvements in both oxygen saturations and end-tidal CO_2_ levels [18]. While this technique is promising, the required technical skill level and equipment limits generalizability in OHCA. Moreover, ultrasound-guided compressions may prove to be of most benefit early on scene with the initiation of CPR.

It is conceivable that lay rescuers and prehospital personnel could be guided to perform compressions over the long axis of the LV with the assistance of a rapid, automated ultrasound device similar to a bladder scanner. Such a device would not require the operator to interpret ultrasound images, but rather would generate a signal informing where compressions should be performed based on automated scanning and calculations. Bladder scanners are non-invasive automated ultrasound devices that can rapidly identify and calculate the volume of a bladder using automated imaging and artificial intelligence (AI). As such, they are used to locate and determine bladder volume by an operator with minimal training without requiring them to perform image interpretation [19, 20]. Though developed to measure bladder volumes, bladder scanners have previously been studied as a potential screening tool to detect abdominal aortic aneurysms with evidence of moderate performance [21, 22]. While a recent study showed the LV is most often located along the left sternal border at the 6th intercostal space (ICS), considerable variability persists with LV location ranging from the 3rd to 7th ICS [23]. Bladder scanners have also been shown to reliably localize the heart in healthy volunteers, with largest scan volumes being most often associated with the area over the long axis of the LV [25]. The performance of a bladder scanner during cardiac arrest, where the heart is no longer beating, remains unknown.

Given the potential for improved outcomes and the association of largest automated scan volumes with the area over the long axis of the LV, the prospect of applying an automated ultrasound device during OHCA to guide chest compressions to the intercostal space over the LV warrants exploration. In this proof-of-concept study, using a porcine model, we describe the performance of a bladder scanner in terms of correctly identifying and tracing borders of the heart in three distinct physiologic states: pre-arrest, arrest, and late arrest.

## Methods

To test our hypothesis, 7 female Landrace swine (Prairie Swine, Saskatoon, SK, CAN) were used in a prospective, repeated measures study. All animal procedures were conducted in accordance with the National Institutes of Health policy on the use of animals in research and approved by the Research Ethics Board Animal Use and Care Committee at the University of Saskatchewan (Saskatoon, SK, CAN; Animal Use Protocol #20200042). This study exploring rapid ultrasonographic identification of the heart was one of several concurrent investigation protocols performed in these swine with these 7 study subjects representing a convenience sample based on compatibility of protocols and resources. Following a minimum 7-day acclimatization, swine were anesthetized, intubated, mechanically ventilated, instrumented, heparinized, and placed supine in a V-shaped holder [25]. Continuous heart rate and cardiac activity were monitored by electrocardiogram (ECG) using a traditional 3-lead limb system (ADI, Colorado Springs, CO, USA).

**Fig. 1:**
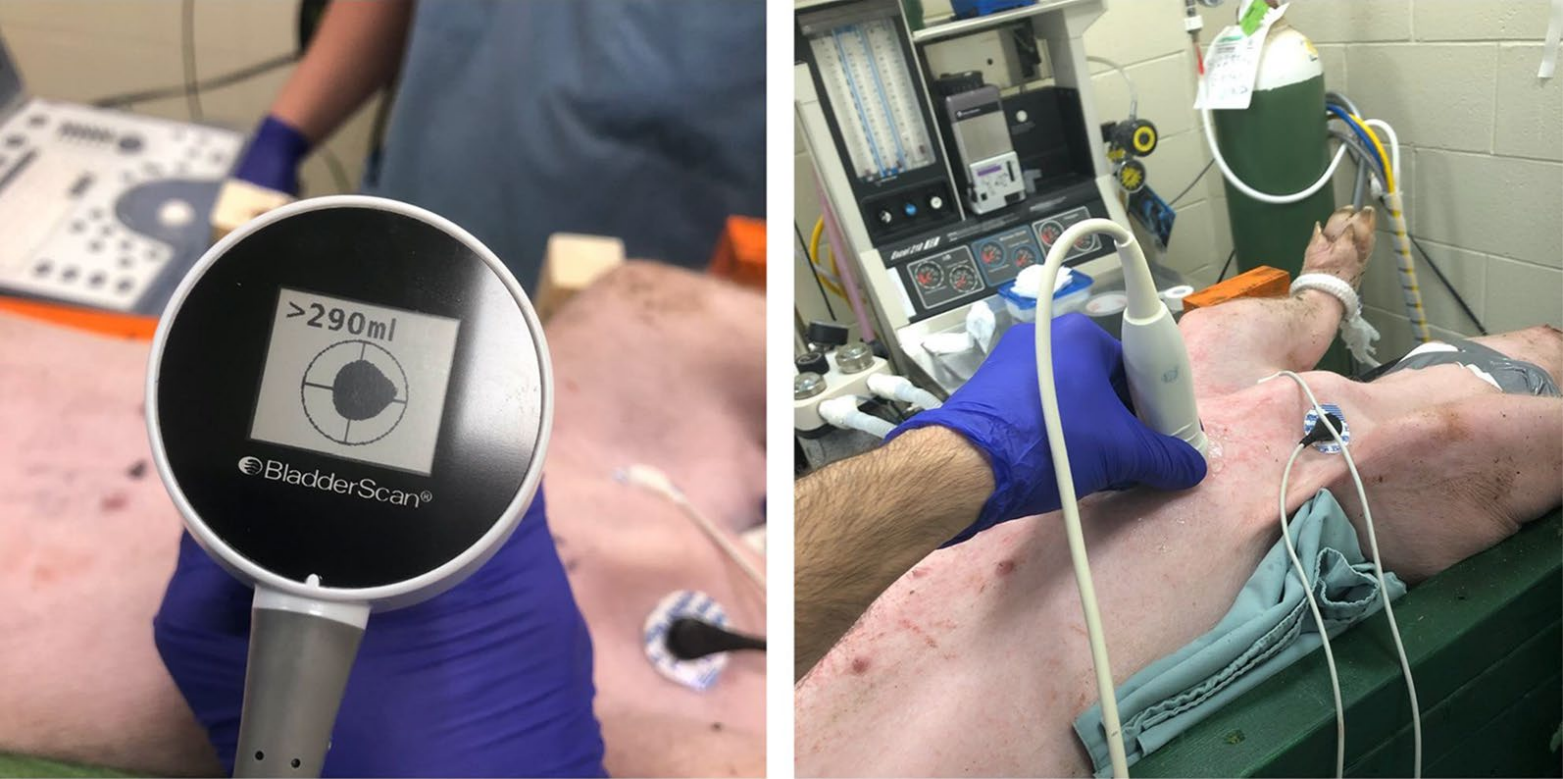
Experimental sonographic localization of the heart with the operator standing on the animal’s left side, the transducer placed perpendicular to the chest wall along the left sternal border. Image on the left shows the transducer is directly over a fluid-filled structure (heart). Echocardiography was performed to describe the underlying cardiac anatomy at each intercostal space (right image)

**Fig. 2.**
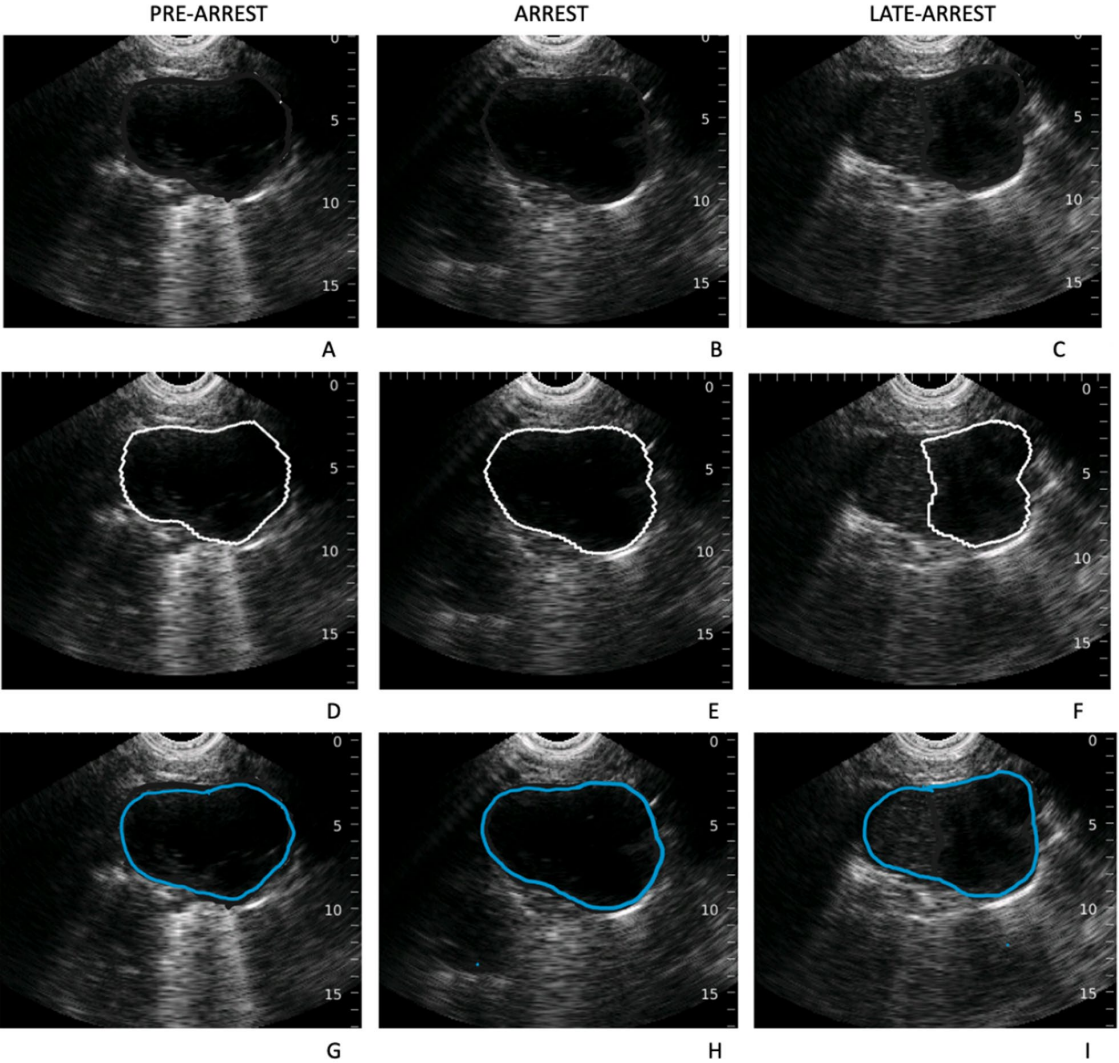
5th intercostal space in pre-arrest (left column), arrest (center column) and late arrest (right column). Top row displaying image capture by the bladder scanner (**a**–**c**, tracing obscured), middle row displaying tracing by bladder scanner’s AI (**d**–**f**), and bottom row showing human reviewer tracing (**g**–**i**). Images **c**, **f**, **i** show an area of echogenicity that the human reviewer interpreted as within the heart, in contrast to the bladder scanner tracing (image F)

Experimental sonographic localization of the heart was performed using a Verathon BladderScan Prime Plus bladder scanner (Verathon, WA, USA). With the operator standing on the animal’s left side, the transducer was placed perpendicular to the chest wall along the left sternal border. Pre-arrest scans were performed from the 4th to the 8th ICSs, with scanner-generated images recorded for each space (see Fig. 1). Echocardiography was then performed by author PO using a GE Vivid i (General Electric, NY, USA) to describe the underlying cardiac anatomy of each animal at each ICS (aortic root, LV outflow tract, LV, and apex). When concurrent study protocols permitted, additional pre-arrest scans were obtained in an effort to increase the number of images available for analysis. Asphyxiated cardiac arrest was then induced and cardiac standstill was confirmed with transthoracic echocardiography (TTE). At the time of arrest, the same ICSs (as described above) were again scanned with the bladder scanner. Finally, when possible within the confines of concurrent protocols, the aforementioned ICSs were scanned during late arrest with several scans performed at each ICS. For each ICS scan, the BladderScan Prime Plus recorded 12 B-mode images (the transducer’s biplane imaging generates 6 orthogonal image pairs). To determine volume, the bladder scanner’s AI generates border tracings which are superimposed on each of the 12 saved images (see Fig. 2). A single scan (12 images) and volume calculation is completed in under 5 s.

**Fig. 3:**
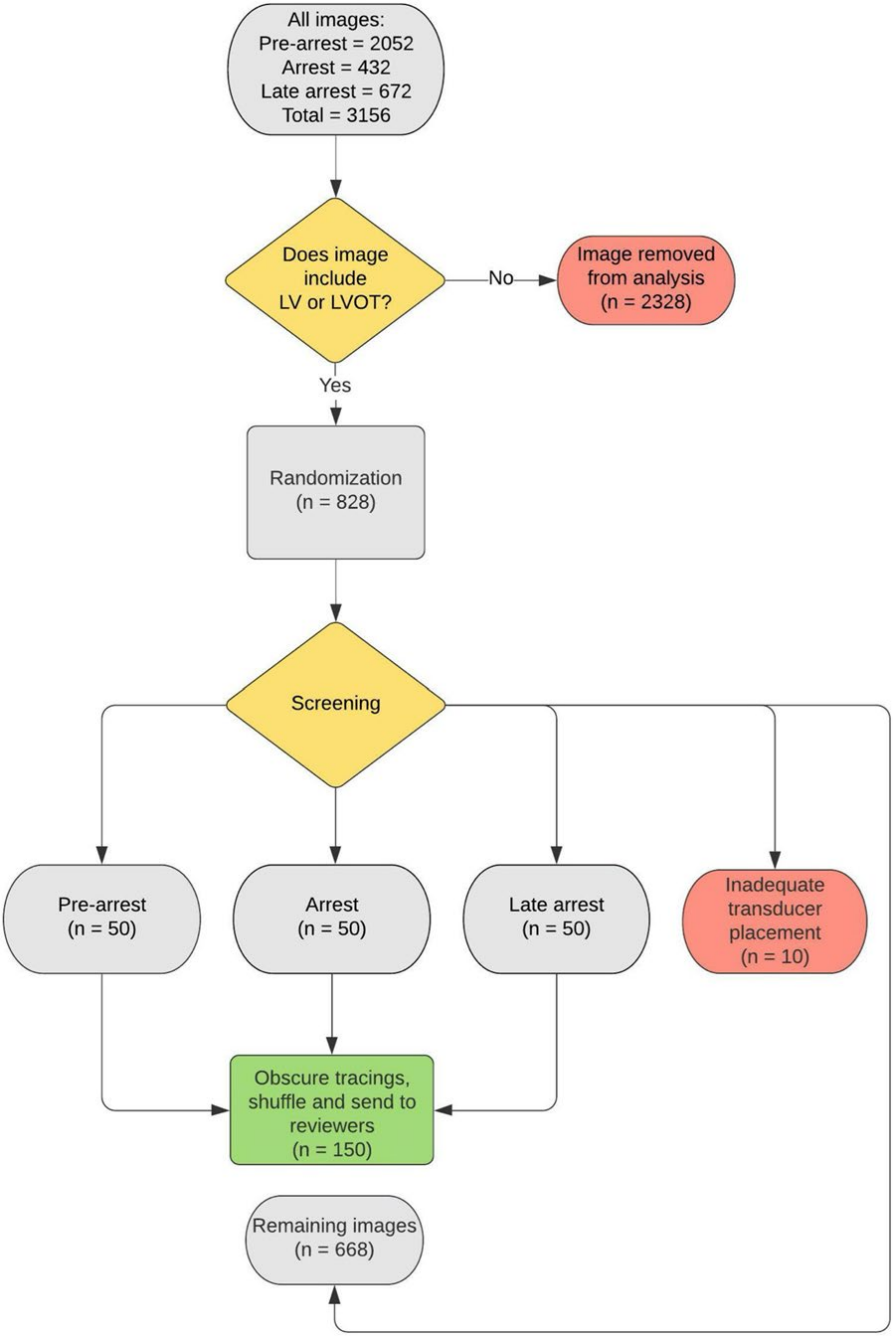
Image selection process: of 3156 images, 828 met criteria for inclusion according to visualization of LV and/or LVOT. Images were randomized and then screened for improper transducer placement (i.e., are of interest obstructed by rib shadow) until there were 50 images for each physiologic state. A total of 160 images were screened, with 10 being excluded due to improper transducer placement. (LV: left ventricle; LVOT: left ventricular outflow tract)

To determine the performance of the bladder scanner’s AI in identifying and tracing a heart, its tracings were compared to 3 expert reviewers certified in focused cardiac ultrasound. The effect that the physiologic state (pre-arrest, arrest and late arrest) had on bladder scanner performance after recording 263 image sets (3156 images captured) was also explored. To approximate the cardiac view as seen along the left sternal border in humans, image analysis was narrowed to those intercostal spaces where the LV or LVOT were identified during TTE. The resulting sample included 69 image sets (828 images) for randomization (see Fig. 3). Assuming a normal distribution of data, we determined 150 images (50 images per state) would provide a sufficient sample (1.97 M pixels/image) while avoiding reviewer fatigue. Images were randomly selected and screened for inclusion as images with significant rib shadows obstructing the view of the heart were excluded (10/160 selected images). The images were coded according to the specific animal, state of arrest, intercostal space, and scan number. These coded images were then shuffled to ensure that each of the human reviewers were not aware of the state of arrest. The tracings created by the bladder scanner were obscured (Adobe Photoshop, CA, USA) to allow for a free trace by the reviewers. Three authors (PA, PO and RW) independently reviewed and then traced their own borders onto the images (Fig. 2, images G-I). The tracings of each human reviewer were compared to those generated by the bladder scan (D-F). Image segmentation analysis between the two sets of images (bladder scanner AI and pooled human reviewers) was determined via Sørensen–Dice coefficients [26]. The Sørensen–Dice index (SDI) is a formula that compares a known-truth sample against an estimated-truth sample. The formula (2 TP/(2 TP + FP + FN)) outputs a single value indicating how closely the two samples match up. A value of 1 represents perfect agreement, and a value of 0 means no agreement.

**Fig. 4:**
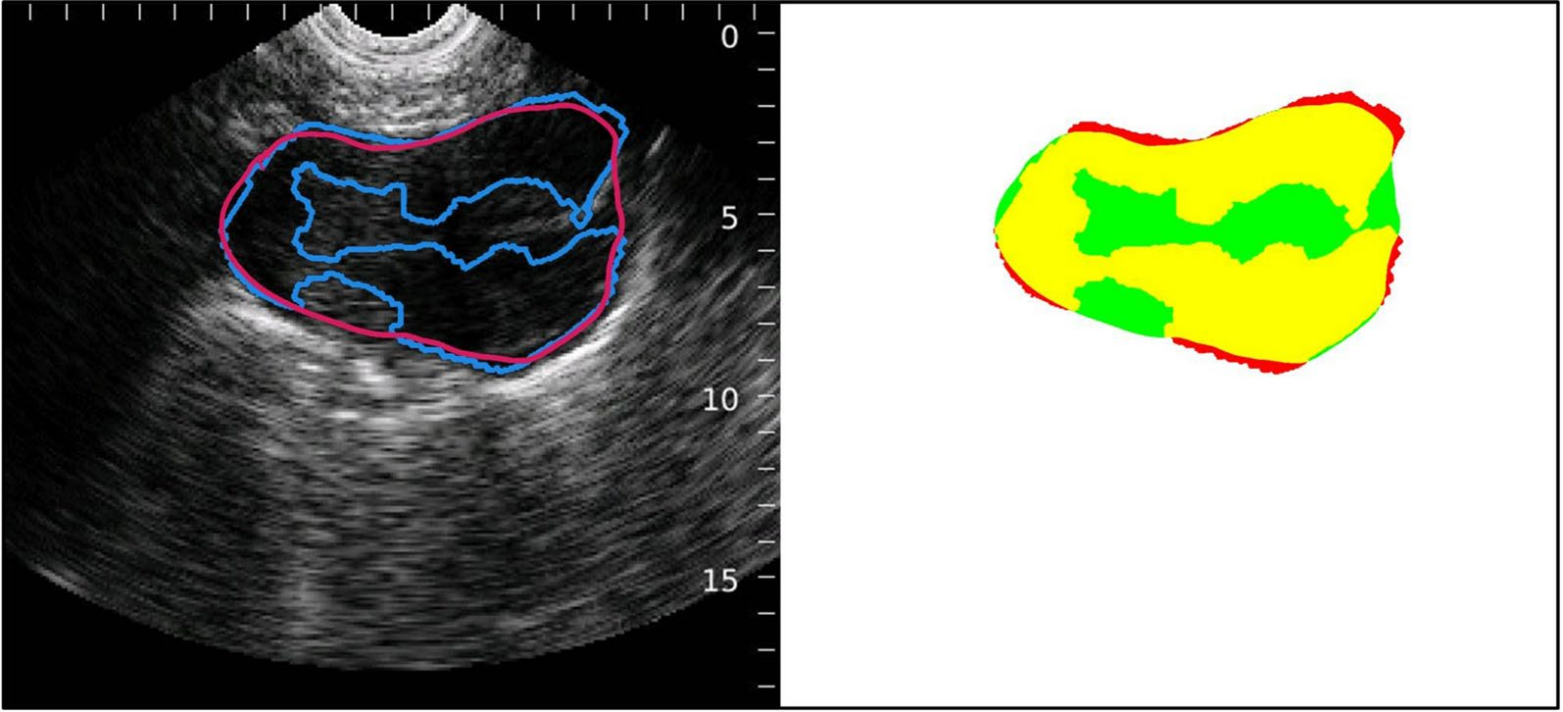
The left shows a single image with both the bladder scanner tracing (blue) and a human reviewer tracing (magenta). The right shows the same image with a visual representation of true positive (yellow, 11.304%), true negative (white, 83.450%), false positive (red, 0.172%) and false negative (green, 5.074%) areas

Reviewer and bladder scanner tracings were processed using software developed and tested by co-author CA [27]. The software was programmed to crop tracings to the bladder scan image area as seen in Fig. 2. Next, the software filled the tracing outline and converted the images to black and white (white = pixel within tracing, black = pixel not within tracing). Finally, the software compared each black and white filled human reviewer tracing against its bladder scanner counterpart pixel-by-pixel to calculate the SDI. The image analysis is represented as true positive (yellow), true negative (white), false positive (red), false negative (green). The overlapping portion captured by the human reviewer and the bladder scanner is shown in yellow. The segment that was captured by the bladder scanner but not the human-reviewer is red, and the segment traced by the human reviewer but was not captured by the bladder scanner is green (see Fig. 4).

## Results

We performed and recorded scans on 7 Landrace swine with masses of 35 ± 2 kg. The mean cardiac mass (post-mortem) was 207.3 g (186–222 g), with the LV most often found over the left sternal border at the 6th intercostal space (range 5–7th). All seven animals underwent pre-arrest and arrest scans in the 4th–8th IC spaces. Owing to concurrent protocols, 5/7 animals attained ROSC during resuscitative efforts and were not eligible for late arrest scans. Late arrest images in 2/7 animals were captured 17- and 32-min post-initial arrest.

The mean SDIs during pre-arrest, arrest, and late arrest were 0.857 (SD 0.157), 0.900 (SD 0.059), and 0.597 (SD 0.241), respectively. SDIs for the performance of the BladderScan Prime as compared to each reviewer for each physiologic state are outlined in Table 1.

**Table 1:** Sørensen–Dice index (SDI) averages for each state of arrest: pre-arrest, arrest, late arrest for each reviewer and a combined average with standard deviations (SD)

	Pre-arrest SDI (50 images)	Arrest SDI (50 images)	Late arrest SDI (50 images)
Reviewer #1	0.887 (0.162)	0.929 (0.041)	0.623 (0.235)
Reviewer #2	0.846 (0.152)	0.900 (0.056)	0.599 (0.239)
Reviewer #3	0.837 (0.151)	0.871 (0.063)	0.570 (0.245)
Mean	0.857 (0.157)	0.900 (0.059)	0.597 (0.241)

## Discussion

In this study, we demonstrate that an automated ultrasound device’s AI was able to identify and trace the borders of porcine hearts. The bladder scanner’s AI performed best at the time of arrest (SDI 0.900 SD 0.059). As resuscitation efforts continued and time from initial arrest increased, the scanner’s performance decreased dramatically (late arrest SDI 0.597 SD 0.241). These findings support our hypothesis that rapid and reliable automated sonographic localization of the heart early in cardiac arrest is possible.

The AI operating within the Verathon BladderScan Prime Plus (Verathon, WA, USA) was developed and taught to identify and trace the border of a bladder, and then determine a volume (based on 6 pairs of biplane images). Challenges presented by a beating heart (where volumes change during the cardiac cycle of contractions and relaxation) explain the decreased performance we noted in the pre-arrest state. Our previous study on the performance of the same bladder scanner on healthy human volunteers showed a strong correlation between the largest scan volume (and thus indirectly the largest tracing) and the area over the long axis of the LV [25]. A major limitation of those findings related to the need for multiple scans (4 per ICS) which was done as a means of capturing the heart near maximal filling (end-diastole). Having to perform several scans with such a device would not be practical during OHCA since delays in compressions have been shown to decrease survival [1]. Since the performance of the scanner is best early in cardiac arrest when the heart has just stopped beating, it is possible that early use of an automated ultrasound device that simultaneously scans several ICSs could efficiently guide compressions to the area over the largest tracing (volume) which is highly associated with the area over the LV. Much like automated electrical defibrators analyze rhythm and guide defibrillation decisions, automated sonographic localization of the left ventricle could guide first-responders’ area of compression over the left ventricle without the need for them to interpret ultrasound images. Instead, such a device would have an indicator directing rescuers to the optimal area of compression after correctly tracing the heart and determining where the LV is based on largest scan volumes (see Fig. 1).

The significant deterioration in performance identified in the late arrest scans led us to review both the bladder scanner and echocardiographic images of the animals. We surmise this is attributable to the effect of low flow and clot formation within the cardiac chambers, occurring despite heparinization. This type of clotting has been reported by Budhram et al. where thrombus occurred within minutes after the induction of ventricular fibrillation (VF) in swine subjects [28]. It is likely that a similar phenomenon happens in cardiac standstill. Both scenarios are relevant to humans suffering OHCA and have implications on the machine learning required for rapid sonographic localization and tracing of the heart. Additionally, the time frame during which the late arrest images displayed clotting within the ventricles (17- and 32-min post-arrest) reinforce the time-sensitive need for rapid and reliable method of LV localization during the initiation of CPR.

The ability of the bladder scanner’s AI to identify the outline of the porcine heart at the time of arrest has been demonstrated. However, the study does have several limitations. As an animal study, questions remain about the performance of such a device on human subjects suffering cardiac arrest. This includes differences in ICS widths that may impact image generation differently in humans, as well as differences in cardiac size and orientation within the thorax. As such, we limited our analysis to images that would be similar to those visualized along the left sternal border in humans (LV and LV outflow tract). This process resulted in images being selected from 2 to 3 ICSs per animal. We also excluded images with significant rib shadowing over the area of interest (due to placement of the transducer over the rib) as this represents operator error attributable to the narrowness of porcine ICSs. The current sample size was small (*n* = 7), and animals were young and healthy with no known intrathoracic disease. Human patients who suffer cardiac arrest have a higher prevalence of cardiac and thoracic pathology, including pleural and pericardial effusions, cardiomyopathies, valve, and vascular abnormalities, having the potential to significantly alter imaging performance [29]. Conversely, because several scans were performed on each animal whenever possible, we did have a significant image set to work from, providing statistically meaningful results. Finally, while we sought to obscure the tracings generated by the bladder scanner, it is possible that these may occasionally have been sufficiently visible to impact human reviewer tracings.

## Conclusion

In this cross-sectional study, an automated ultrasound device (bladder scanner) was able to reliably identify and trace the borders of porcine hearts at the time of cardiac arrest. Bladder scanner performance was best during the initial arrest state and deteriorated significantly in late arrest likely due to low flow and clotting in the cardiac chambers. Further investigation into rapid sonographic localization of the heart to identify the area of LV compression in OHCA is warranted.

## Data Availability

The datasets used and/or analyzed during the current study are available from the corresponding author on reasonable request.
